# Integrating active and passive digital phenotyping to study the temporal dynamics between physical activity and mood in bipolar disorders

**DOI:** 10.1038/s44277-025-00050-z

**Published:** 2025-12-02

**Authors:** Audrey R. Stromberg, Amy Bohnert, Srijan Sen, Sarah H. Sperry

**Affiliations:** 1 Department of Psychiatry, University of Michigan, Ann Arbor, MI USA; 2 Department of Anesthesiology, University of Michigan, Ann Arbor, MI USA

**Keywords:** Bipolar disorder, Human behaviour

## Abstract

Bipolar disorders (BDs) are characterized by pronounced mood fluctuations that can impair daily function and treatment. While long-term benefits of physical activity for mood are well-established, less is known about how day-to-day changes in activity impact mood in individuals with BDs. This study leverages 12 months of digital phenotyping and ecological momentary assessment data from the University of Michigan’s PROviding Mental Health Precision Treatment study to examine day-level associations between mood, physical activity (steps, very active minutes), and resting heart rate (RHR) in 252 adults with BDs. Participants completed daily mood ratings via smartphone (prompted at 6:00PM) and wore Fitbits that passively recorded steps, very active minutes, and RHR (available on days with ≥30 min of still periods). Using Dynamic Structural Equation Modeling, we observed a small but credible bidirectional relationship between daily steps and mood: individuals who reported better mood than their own average took more steps the following day, and vice versa. Very active minutes predicted next-day mood but not the reverse, which—while possibly due to their low frequency in the sample—may suggest that higher-intensity activity does not produce a feedback loop similar to total steps. No credible cross-lag associations emerged between daily RHR and mood. Conditional models found that sex and age influenced several activity dynamics, and baseline anxiety was positively associated with daily RHR variability. These findings highlight that increases in physical activity, especially in daily steps, may support mood regulation in BDs on the day level.

## Lay Summary

This study explored how daily physical activity impacts mood in people with bipolar disorder over one year. Using Fitbit devices and daily mood surveys, we found that having a better mood usually led to taking more steps the next day—and vice versa. More intense activity improved next-day mood, but not vice versa. Resting heart rate changes weren’t linked to mood. Overall, regular physical activity, especially daily steps, may help with mood in bipolar disorder.

## Introduction

Nearly 4% of the population is diagnosed with a bipolar spectrum disorder (BD) [1], a condition that results in significant functional impairment [2] and increased mortality risk [3]. Despite its public health impact, etiological and maintenance factors of BD remain unclear, contributing to ongoing challenges in developing effective interventions. Increasingly, research indicates that physical activity may impact BD symptoms [4]. Understanding the interplay between physical activity and mood in individuals with BDs may reveal patterns that could inform treatment interventions.

It is well established that activity levels impact both physical and mental health. Reducing sedentary behavior and increasing physical activity helps prevent cardiovascular disease, diabetes, and metabolic syndromes [5], all of which are comorbidities commonly reported in BDs [6]. Given individuals with BDs may be prone to lower activity levels [7, 8], it is especially important to study the relationships between activity and physical health in this population.

Research also finds an inverse relationship between daily activity level and mental health symptoms like depression and anxiety [9–12]. These associations are also present in BD, where cross-sectional and longitudinal evidence suggests that increased activity levels mitigate symptoms of bipolar depression [6, 13, 14]. Recent work in this area suggests that improvements in mood derived from physical activity may occur as soon as next-day. For example, Merikangas and colleagues (2019) found that, over two weeks, increased motor activity was associated with better mood at the next time period, particularly for individuals with BD I [15]. Similarly, increased vigorous activity was associated with reduced next-day depressive symptoms in 28 individuals with BD over 20 days [16]. Together, these studies show that physical activity may confer mood benefits at the day level in BD, while highlighting the need to replicate these findings in larger, longer-term studies.

Additionally, growing evidence suggests individuals with BDs may differ from non-psychiatric populations in the function of their autonomic nervous system (ANS) [17, 18], which regulates involuntary physiological processes, including heart rate [19]. While much research relies on heart rate variability (HRV) as a proxy of ANS function, another easily accessible index of ANS activity is resting heart rate (RHR). In both psychiatric and non-psychiatric populations, higher RHRs have been linked with all-cause mortality [20, 21] and higher risk of suicide, an outcome commonly linked with severe depressive symptoms [22–24]. Furthermore, there is evidence that for some individuals, particularly those with psychiatric symptoms, RHR associates with mood state [25]. Despite these associations, research investigating RHR in BD populations remains limited, particularly regarding how RHR influences more proximal outcomes (e.g., daily mood), despite the elevated suicide and depression risk in this group.

Although long-term associations between activity and mood in BDs are well-established, day-to-day dynamics remain unclear. Most research relies on short-term (e.g., 2-week) or cross-sectional studies with small samples, limiting analysis of temporal fluctuations typical in BDs. Since mood instability is central to BDs [26, 27], examining daily predictors and outcomes of mood shifts in larger longitudinal studies could offer valuable insights for treatment.

Digital phenotyping offers a promising approach to address this gap, allowing for day-to-day measurement of mood, RHR, and objective physical activity [28, 29]. While still relatively new in BD research, digital phenotyping holds promise for disentangling complex relationships between variables as they unfold in daily life. For example, Lipschitz and colleagues (2024) applied machine learning models to Fitbit data and found that RHR and “very active minutes” (i.e., exercise minutes classified as higher intensity based on HR) were among the variables with highest relative importance in the algorithms predicting depressive and (hypo)manic episodes, respectively [30]. However, machine learning algorithms are designed for prediction rather than inferential analysis. Thus, while these findings highlight the importance of metrics such as very active minutes and RHR in relation to mood, they leave open questions about the temporal relationships with mood dynamics over time, like how changes in steps, exercise intensity, and RHR dynamically impact mood at the day-level. These types of questions are better addressed by inferential methods like Dynamic Structural Equation Modeling (DSEM) [31].

The purpose of the present investigation is to examine the day-to-day dynamics between physical activity (i.e., daily steps, very active minutes), RHR, and mood in BDs using a large longitudinal dataset of active and passive ambulatory assessment data. We leveraged data from the University of Michigan’s PROviding Mental Health Precision Treatment (PROMPT) [32] study to test the following hypotheses: 1) Within-person increases in daily steps will be associated with better (i.e., increased) mood and vice versa; 2) Within-person increases in minutes classified as “very active” (i.e., higher intensity based on metabolic equivalent of task or MET) will be associated with better mood and vice versa; 3) Within-person decreases in RHR would be associated with better mood.

## Method

The PROMPT study follows participants for one year. For the purposes of this study, we extracted data collected from May 13, 2020 to August 13, 2023. All participants provided written informed consent, and the study received approval from the University of Michigan Institutional Review Board. While not the focus of the present analysis, participants were randomized to one of 5 low-intensity digital intervention groups: 1) enhanced personalized feedback (EPF), 2) Silvercloud (American Well Corporation), a mobile application that provides CBT strategies, 3) Silvercloud plus EPF, 4) Headspace, a mobile application that provides mindfulness strategies, and 5) Headspace plus EPF (see Supplement Appendix 1 for more details on the PROMPT protocol). The study adhered to Consolidated Standards of Reporting Trials (CONSORT) guidelines.

### Setting and participants

Study participants were adults on the waitlist to receive outpatient psychiatric services at various mental and behavioral health clinics. Most participants were recruited from outpatient psychiatry clinics, with fewer from campus and primary care clinics. Eligible participants were English-speaking adults aged 18 or older who either had an upcoming mental health appointment or had completed an initial appointment within the last 60 days. For the present investigation, only participants who had a chart diagnosis of a BD were included. Participants were required to have daily access to a smartphone. Exclusion criteria included cognitive impairments or guardianship issues that prevented informed consent, as well as active eating disorders which, according to the judgment of patients or staff, would make the use of an activity tracking device inappropriate. Self-reported race and ethnicity data were collected.

### Study design and procedures

Study staff contacted potentially eligible patients by phone, text, or email. After enrollment, participants completed a baseline survey that included demographic and clinical assessments using a health application (MyDataHelps). They used a study activity tracking device (Fitbit or Apple Watch), and they linked their smartphone and wearable device to MyDataHelps. The present investigation focuses exclusively on Fitbit data due to the limited number of Apple Watch users and the significant differences in data formats between the two devices, which made integration into a single dataset problematic.

Participants underwent 12 months of continuous digital phenotyping using Fitbit and EMA using MyDataHelps. Throughout the study, research staff monitored for noncompliance and contacted participants via text or email when issues were identified, providing support to resolve any technical difficulties. Reminders were also issued through notifications from MyDataHelps. Participants were compensated for baseline and follow-up assessments.

### Materials and procedures

#### Self-report items

##### Mood

The primary outcome of this study was a one-item EMA mood assessment, which prompted participants at 6:00PM to rate their average mood for the day on a scale from 1 (worst mood) to 10 (best mood) [33, 34]. This was reported daily for the full study duration.

##### Patient health questionnaire (PHQ-9)

Depression was measured at baseline using the PHQ-9 [35], which assesses depressive symptoms over the past two weeks. Scores range from 0 (no depression) to 27 (severe depression).

##### Generalized anxiety disorder scale (GAD-7)

Anxiety was measured at baseline using the GAD-7 [36], which assesses generalized anxiety over the past two weeks. Scores range from 0 (no anxiety) to 21 (severe anxiety).

#### Fitbit measures

We examined four standard measures derived from Fitbit: daily steps, very active minutes, RHR, and total sleep time (TST). Please see Table 1 for details and definitions of these measures.

**Table 1:** Fitbit Measures and Definitions.

Measure	Definition
Daily steps	Daily steps were recorded as the total number of steps taken by participants each day, with higher values indicating greater physical activity.
Very active minutes	Fitbit calculated daily minutes spent in vigorous physical activity, defined as periods of activity with a metabolic equivalent of task (MET) value greater than 6.0, a common reference threshold of vigorous activity [48]. Notably, while Fitbit’s estimates of energy expenditure and heart rate zones are dependent on person-specific information (e.g., age, weight, sex), the MET threshold used to classify very active minutes is consistent across all participants.
Resting heart rate (RHR)	RHR was defined as the average number of heart beats per minute during periods of sleep and inactivity during the day, which was used as an indicator of cardiovascular health. Fitbit requires at least 30 min of still periods (including daytime wear) to calculate this metric [49]. This was measured daily.
Total sleep time (TST)	TST reflects total minutes asleep during the main sleep period, excluding wake time after initial sleep onset.

All of the following metrics reflect standard measures provided by Fitbit.

### Data pre-processing

To ensure data quality, the Fitbit data were cleaned and pre-processed before analysis, resulting in 252 individuals with sufficient data for inclusion. The final dataset only included participants who contributed at least 30 days of data (suiting the requirements of DSEM) during which the Fitbit was worn for at least 10 h of waking wear, in line with best practices [37]. Across the entire dataset, a total of 32,451entries were recorded. On average, participants contributed 125.92 days of Fitbit data (range = 30–347; median = 105; SD = 79.91). For mood EMA data, participants contributed an average of 128.77 days (range = 30–348; median = 108; SD = 81.15). Daily step counts were log-transformed to reduce variability, which was initially too high for effective modeling in DSEM, meaning all coefficients involving daily steps should be interpreted on a proportional scale. Please see Supplement (Appendix 2) for additional details on data cleaning, adherence requirements, and pre-processing steps.

### Statistical analysis

Within- and between-person correlations (Spearman’s rho; Supplement Figure S1) between daily mood, steps, very active minutes, and RHR were estimated using functions from the ‘psych’ package in RStudio (R Version 4.4.3). To model the temporal dynamics between mood and the Fitbit measures, we used DSEM. DSEM is a Bayesian multivariable multilevel first-order vector autoregressive model estimated in MPlus v8.8 [31]. Comprehensive details regarding this modeling approach are provided in Supplement (Appendix 3). Three unconditional models were fit for each predictor (steps, very active minutes, RHR). These models resulted in eight random effects: mean intensity, autoregressive coefficients (i.e., how much a variable predicted itself at the next time point), cross-lagged regressions (i.e., bi-directional relationships, meaning how much a variable predicted another variable at the next time point), and innovation variance (i.e., the leftover “noise” or variability in the time series of the variable after accounting for autoregression and cross-lagged effects). All continuous within-person variables are standardized by Mplus in DSEM, meaning coefficients represent standardized effects. Conditional models were run to examine whether age at entry, sex, baseline depression and anxiety (PHQ-9 and GAD-7 score), or randomization group predicted each of these 8 random effects. Randomization groups were added as covariates to account for the PROMPT’s intervention design, assessing whether the examined dynamics were affected by treatment condition, compared to those in the EPF-only condition (Group 1; reference group). Results were evaluated based on the 95% credible interval, where intervals not containing zero were considered credible differences or effects.

To address potential violations of stationarity over the study period, a within-person “Time” predictor with random slopes for each outcome was included in the DSEM models. Additionally, to address the potential effect of sleep on mood, the prior night’s TST was included in all conditional models to assess the effect of prior night’s TST on daily mood.

## Results

### Participants

The present study included 252 participants (74.6% female) with a BD diagnosis in their electronic health record (EHR). The average age at study entry was 40.37 years (range = 18.43 – 76.12; median = 37.12; SD = 14.45), and racially the sample was 79.0% White, 7.1% Black or African-American, 5.2% Asian, 2.8% American Indian or Alaska Native, and 2.8% other race (Table S1).

### Activity level

Participants averaged 5984 (median = 5444) steps and 11.2 (median = 5.3) very active minutes per day (see Supplement Tables S2–S3 for full descriptives). Based on the U.S. Department of Health and Human Services guidelines, these combined activity levels suggest that participants in this sample met or exceeded national recommendations for weekly aerobic activity [38, 39]. Notably, activity and fitness levels varied across participants (see Supplement Table S4 for descriptives by age and sex).

### Time

In the models with daily steps and very active minutes, Time slopes were statistically credible but small. At the within-person level, daily mood increased slightly over time (estimate = 0.03; small effect), and daily steps decreased slightly over time (estimate = −0.05; small effect). With Time in these models, autoregressive and cross-lagged associations remained robust, mitigating concerns about violations of stationarity. The effects of Time were not credible in the RHR model.

### Longitudinal patterns of daily mood

Across all models, the autoregressive coefficients for mood, the extent to which an increase in mood at one time point predicts an increase at the next time point, were credible (estimates = 0.33 to 0.34). In all conditional models (daily steps, very active minutes, RHR; Tables 2–4), older age was associated with a stronger autoregressive effect of daily mood (estimates = 0.21 to 0.22; small-medium effects), while younger age was associated with increased variability in daily mood (estimates = −0.20 to −0.22, small-medium effects).

**Table 2:** Conditional Mood and Log Steps.

		Between Subjects Predictors of Random Effects							
		Depression	Anxiety	Sex	Age	Group 2	Group 3	Group 4	Group 5
Variable	Estimated Mean [95% CI]	Estimate [95% CI]	Estimate [95% CI]	Estimate [95% CI]	Estimate [95% CI]	Estimate [95% CI]	Estimate [95% CI]	Estimate [95% CI]	Estimate [95% CI]
Mood mean intensity $({\mu }_{{Mood}})$	**6.09 [5.65, 6.49]**	−**0.22 [**−**0.34, -0.08]**	**−0.13 [−0.27, −0.01]**	0.01 **[−**0.09, 0.11]	**−**0.02 **[−**0.12, 0.08]	**−**0.01 **[−**0.11, 0.10]	**−**0.03 **[−**0.12, 0.07]	**−**0.03 **[−**0.15, 0.10]	0.01 **[−**0.11, 0.11]
Log steps mean intensity $({\mu }_{{Stepslog}})$	**8.45 [8.28, 8.65]**	**−**0.04 **[−**0.16, 0.10]	0.01 **[−**0.12, 0.13]	**0.22 [0.13, 0.31]**	**−0.13 [−0.22, −0.02]**	**−**0.02 **[−**0.13, 0.08]	**−**0.01 **[−**0.12, 0.08]	0.01 **[−**0.11, 0.12]	**−**0.03 **[−**0.14, 0.07]
Mood Autoregression $({\phi }_{{MM}})$	**0.33 [0.28, 0.38]**	**−0.13 [−0.25, 0.00]**	0.09 **[−**0.04, 0.21]	**−**0.02 **[−**0.13, 0.08]	**0.22 [0.12, 0.31]**	0.01 **[−**0.09, 0.12]	**−**0.07 **[−**0.17, 0.03]	0.04 **[−**0.09, 0.14]	**−**0.04 **[−**0.15, 0.07]
Log steps Autoregression $({\phi }_{{SS}})$	**0.21 [0.16, 0.26]**	0.05 **[−**0.10, 0.19]	0.05 **[−**0.10, 0.21]	**−**0.01 **[−**0.12, 0.10]	**−**0.04 **[−**0.16, 0.07]	0.02 **[−**0.11, 0.14]	**−**0.05 **[−**0.17, 0.06]	0.04 **[−**0.09, 0.17]	**−**0.06 **[−**0.18, 0.06]
Mood_*t*-*1*_ → Log steps_*t*_ $({\phi }_{{MS}})$	**0.02 [0.01, 0.04]**	**−**0.10 **[−**0.32, 0.14]	**−**0.06 **[−**0.29, 0.17]	0.15 **[−**0.04, 0.33]	**−**0.06 **[−**0.25, 0.12]	**−**0.05 **[−**0.24, 0.14]	**−**0.03 **[−**0.23, 0.18]	**−0.22 [−0.42, −0.01]**	**−**0.09 **[−**0.27, 0.08]
Log steps_*t*-*1*_ → Mood_*t*_ $({\phi }_{{SM}})$	**0.08 [0.03, 0.13]**	0.06 **[−**0.16, 0.26]	**−**0.10 **[−**0.32, 0.12]	0.01 **[−**0.14, 0.16]	**−**0.06 **[−**0.25, 0.11]	**−**0.04 **[−**0.24, 0.14]	0.03 **[−**0.13, 0.20]	0.05 **[−**0.14, 0.25]	0.00 **[−**0.18, 0.20]
Mood variability (${\log} ({\pi}_{Mood})$)	0.22 **[−**0.00, 0.41]	**0.14 [0.02, 0.25]**	0.02 **[−**0.08, 0.15]	**−**0.04 **[−**0.13, 0.04]	**−0.20 [−0.28, −0.11]**	**−**0.03 **[−**0.12, 0.07]	**−**0.09 **[−**0.18, 0.00]	**−**0.02 **[−**0.12, 0.09]	**−**0.01 **[−**0.12, 0.08]
Log steps variability (${\log } (\pi_{{Stepslog}})$)	**−1.07 [−1.22, −0.92]**	0.11 **[−**0.01, 0.23]	**−**0.04 **[−**0.15, 0.08]	**−**0.07 **[−**0.16, 0.02]	**−**0.07 **[−**0.16, 0.03]	**−**0.07 **[−**0.17, 0.03]	0.00 **[−**0.10, 0.09]	0.03 **[−**0.08, 0.14]	**−**0.06 **[−**0.16, 0.03]
Mood over time ($\beta$_*TimeM*_)	0.00 **[−**0.00, 0.00]	0.04 **[−**0.09, 0.18]	0.06 **[−**0.08, 0.19]	**−**0.01 **[−**0.11, 0.09]	0.01 **[−**0.10, 0.11]	**−**0.01 **[−**0.13, 0.10]	0.02 **[−**0.09, 0.13]	0.02 **[−**0.11, 0.13]	0.03 **[−**0.09, 0.14]
Log steps over time ($\beta$_*TimeS*_)	0.00 **[−**0.00, 0.00]	0.07 **[−**0.05, 0.20]	**−**0.08 **[−**0.21, 0.04]	**−**0.05 **[−**0.15, 0.05]	**−**0.00 **[−**0.11, 0.10]	0.01 **[−**0.10, 0.12]	0.05 **[−**0.06, 0.16]	**−**0.02 **[−**0.13, 0.09]	**−**0.08 **[−**0.19, 0.03]
TST on mood ($\beta$_*TSTM*_)	0.00 **[−**0.00, 0.00]	0.03 **[−**0.10, 0.18]	0.03 **[−**0.12, 0.17]	**−**0.03 **[−**0.14, 0.08]	0.01 **[−**0.10, 0.12]	**−**0.02 **[−**0.15, 0.10]	**−**0.01 **[−**0.12, 0.09]	0.01 **[−**0.13, 0.15]	**−**0.03 **[−**0.15, 0.09]
TST on log steps ($\beta$_*TSTS*_)	0.00 **[−**0.00, 0.00]	**−**0.07 **[−**0.20, 0.06]	0.03 **[−**0.12, 0.16]	**−**0.08 **[−**0.19, 0.02]	0.03 **[−**0.07, 0.13]	0.01 **[−**0.11, 0.12]	0.01 **[−**0.10, 0.11]	**−**0.01 **[−**0.13, 0.12]	0.05 **[−**0.07, 0.17]

*CI* Credibility interval. *TST* prior night’s total sleep time. *Group 1 (reference)* enhanced personalized feedback (EPF) only. *Group 2* Headspace only. *Group 3* Headspace + EPF. *Group 4* SilverCloud only. *Group 5* SilverCloud + EPF.

**Table 3:** Conditional Mood and Minutes Very Active (MVA).

		Between Subjects Predictors of Random Effects					
		Sex	Age	Group 2	Group 3	Group 4	Group 5
Variable	Estimated Mean [95% CI]	Estimate [95% CI]	Estimate [95% CI]	Estimate [95% CI]	Estimate [95% CI]	Estimate [95% CI]	Estimate [95% CI]
Mood mean intensity $({\mu }_{{Mood}})$	**5.96 [5.56, 6.35]**	0.05 **[−**0.06, 0.14]	0.06 **[−**0.04, 0.16]	**−**0.00 **[−**0.12, 0.11]	**−**0.02 **[−**0.11, 0.09]	**−**0.03 **[−**0.16, 0.09]	0.01 **[−**0.10, 0.13]
MVA mean intensity $({\mu }_{{MVA}})$	**3.75 [2.02, 5.79]**	**0.39 [0.27, 0.50]**	**−**0.10 **[−**0.20, 0.01]	0.07 **[−**0.05, 0.18]	0.05 **[−**0.07, 0.16]	0.02 **[−**0.10, 0.14]	0.07 **[−**0.05, 0.19]
Mood Autoregression $({\phi }_{{MM}})$	**0.33 [0.28, 0.39]**	**−**0.02 **[−**0.12, 0.08]	**0.21 [0.10, 0.31]**	0.00 **[−**0.11, 0.11]	**−**0.09 **[−**0.19, 0.02]	0.02 **[−**0.10, 0.14]	**−**0.07 **[−**0.18, 0.05]
MVA Autoregression $({\phi }_{{AA}})$	**0.11 [0.05, 0.17]**	**0.10 [0.01, 0.19]**	**−0.11 [−0.20, −0.01]**	**−**0.03 **[−**0.16, 0.08]	**−**0.05 **[−**0.15, 0.06]	**−**0.02 **[−**0.14, 0.10]	**−**0.06 **[−**0.17, 0.05]
Mood_*t*-*1*_ → MVA_*t*_ $({\phi }_{{MA}})$	0.03 **[−**0.04, 0.11]	**0.91 [0.30, 0.99]**	0.07 **[−**0.15, 0.39]	**−**0.04 **[−**0.19, 0.16]	**−**0.14 **[−**0.42, 0.29]	**−**0.06 **[−**0.27, 0.13]	**−**0.02 **[−**0.20, 0.14]
MVA_*t*-*1*_ → Mood_*t*_ $({\phi }_{{AM}})$	0.00 **[−**0.00, 0.01]	**−**0.02 **[−**0.12, 0.09]	**−**0.00 **[−**0.14, 0.12]	**−**0.04 **[−**0.17, 0.09]	**−**0.01 **[−**0.13, 0.11]	**−**0.03 **[−**0.18, 0.11]	**−**0.02 **[−**0.16, 0.12]
Mood variability (${\log } (\pi_{{Mood}})$)	**0.23 [0.01, 0.43]**	**−**0.06 **[−**0.15, 0.03]	**−0.22 [−0.30, −0.14]**	**−**0.03 **[−**0.13, 0.06]	**−**0.09 **[−**0.18, 0.01]	**−**0.02 **[−**0.11, 0.09]	**−**0.02 **[−**0.11, 0.08]
MVA variability (${\log } (\pi_{{MVA}})$)	**3.91 [3.40, 4.37]**	**0.26 [0.17, 0.34]**	**−0.13 [−0.22, −0.04]**	0.01 **[−**0.08, 0.10]	0.04 **[−**0.05, 0.13]	**−**0.01 **[−**0.12, 0.08]	**−**0.02 **[−**0.11, 0.08]
Mood over time ($\beta$_*TimeM*_)	0.00 **[−**0.00, 0.00]	**−**0.02 **[−**0.12, 0.08]	**−**0.02 **[−**0.12, 0.08]	**−**0.01 **[−**0.12, 0.11]	0.03 **[−**0.08, 0.14]	0.02 **[−**0.10, 13]	0.03 **[−**0.08, 0.14]
MVA over time ($\beta$_*TimeMVA*_)	**−**0.00 **[−**0.01, 0.01]	0.04 **[−**0.09, 0.17]	**−**0.09 **[−**0.20, 0.02]	**−**0.06 **[−**0.20, 0.07]	**−**0.01 **[−**0.14, 0.13]	**−**0.04 **[−**0.17, 0.08]	0.03 **[−**0.10, 0.18]
TST on mood ($\beta$_*TSTM*_)	0.00 **[**0.00, 0.00]	**−**0.04 **[−**0.14, 0.07]	**−**0.01 **[−**0.12, 0.10]	**−**0.01 **[−**0.13, 0.12]	**−**0.01 **[−**0.12, 0.10]	0.03 **[−**0.10, 0.16]	0.00 **[−**0.12, 0.12]
TST on MVA ($\beta$_*TSTMVA*_)	**0.01 [0.00, 0.01]**	0.10 **[−**0.02, 0.20]	**−**0.05 **[−**0.15, 0.06]	**−**0.01 **[−**0.13, 0.10]	0.00 **[−**0.11, 0.11]	**−**0.03 **[−**0.15, 0.09]	**−**0.04 **[−**0.15, 0.09]

The $\beta$_*TSTMVA*_ estimate technically reached the threshold of statistical credibility but represents a negligible effect size with a 95% CI spanning zero to the second decimal place and was therefore not interpreted further in the text.

*CI* Credibility interval. *TST* prior night’s total sleep time. *Group 1 (reference)* enhanced personalized feedback (EPF) only. *Group 2* Headspace only. *Group 3* Headspace + EPF. *Group 4* SilverCloud only. *Group 5* SilverCloud + EPF.

**Table 4:** Conditional Mood and Resting Heart Rate (RHR).

		Between Subjects Predictors of Random Effects							
		Depression	Anxiety	Sex	Age	Group 2	Group 3	Group 4	Group 5
Variable	Estimated Mean [95% CI]	Estimate [95% CI]	Estimate [95% CI]	Estimate [95% CI]	Estimate [95% CI]	Estimate [95% CI]	Estimate [95% CI]	Estimate [95% CI]	Estimate [95% CI]
Mood mean intensity $({\mu }_{{Mood}})$	**6.10 [5.71, 6.48]**	**−0.21 [−0.34, −0.08]**	**−0.14 [−0.27, −0.01]**	0.01 **[−**0.10, 0.10]	**−**0.01 **[−**0.11, 0.10]	**−**0.01 **[−**0.12, 0.10]	**−**0.04 **[−**0.14, 0.06]	**−**0.05 **[−**0.16, 0.07]	**−**0.01 **[−**0.12, 0.10]
RHR mean intensity $({\mu }_{{RHR}})$	**73.83 [70.78, 76.74]**	0.03 **[−**0.11, 0.17]	**−**0.01 **[−**0.14, 0.13]	**−**0.03 **[−**0.13, 0.07]	**−**0.07 **[−**0.17, 0.04]	**−**0.01 **[−**0.14, 0.11]	0.09 **[−**0.02, 0.20]	0.02 **[−**0.10, 0.15]	0.08 **[−**0.03, 0.20]
Mood Autoregression $({\phi }_{{MM}})$	**0.33 [0.28, 0.38]**	**−**0.12 **[−**0.24, 0.01]	0.09 **[−**0.04, 0.22]	**−**0.03 **[−**0.13, 0.07]	**0.22 [0.12, 0.32]**	0.01 **[−**0.11, 0.12]	**−**0.08 **[−**0.18, 0.03]	0.02 **[−**0.09, 0.14]	**−**0.06 **[−**0.16, 0.06]
RHR Autoregression $({\phi }_{{RR}})$	**0.89 [0.87, 0.90]**	**−**0.02 **[−**0.17, 0.13]	0.13 **[−**0.02, 0.27]	**−**0.09 **[−**0.20, 0.02]	**−0.15 [−0.26, −0.03]**	0.02 **[−**0.11, 0.14]	0.03 **[−**0.09, 0.16]	0.00 **[−**0.13, 0.13]	**−**0.07 **[−**0.20, 0.05]
Mood_*t*-*1*_ → RHR_*t*_ $({\phi }_{{MR}})$	0.01 **[−**0.02, 0.03]	0.09 **[−**0.19, 0.36]	**−**0.12 **[−**0.40, 0.16]	0.04 **[−**0.17, 0.28]	**−0.24 [−0.57, −0.01]**	0.05 **[−**0.18, 0.27]	0.07 **[−**0.18, 0.30]	**−**0.11 **[−**0.36, 0.13]	**−**0.07 **[−**0.30, 0.15]
RHR_*t*-*1*_ → Mood_*t*_ $({\phi }_{{RM}})$	0.00 **[−**0.01, 0.01]	0.04 **[−**0.13, 0.22]	0.02 **[−**0.18, 0.20]	0.04 **[−**0.11, 0.18]	**−**0.04 **[−**0.18, 0.11]	**−**0.05 **[−**0.20, 0.11]	**−**0.07 **[−**0.21, 0.07]	**−**0.12 **[−**0.28, 0.06]	**−**0.14 **[−**0.31, 0.02]
Mood variability (${\log } (\pi_{{Mood}})$)	**0.22 [0.02, 0.43]**	**0.13 [0.01, 0.24]**	0.03 **[−**0.09, 0.15]	**−**0.04 **[−**0.13, 0.04]	**−0.20 [−0.28, −0.11]**	**−**0.03 **[−**0.12, 0.07]	**−**0.09 **[−**0.18, 0.01]	**−**0.02 **[−**0.12, 0.08]	**−**0.01 **[−**0.12, 0.09]
RHR variability (${\log } (\pi_{{RHR}})$)	**0.56 [0.47, 0.65]**	**−**0.01 **[−**0.13, 0.12]	**0.15 [0.02, 0.27]**	**−**0.05 **[−**0.15, 0.04]	**−**0.06 **[−**0.16, 0.04]	0.03 **[−**0.08, 0.04]	0.07 **[−**0.03, 0.17]	0.08 **[−**0.04, 0.19]	**−**0.01 **[−**0.12, 0.09]
Mood over time ($\beta$_*TimeM*_)	0.00 **[−**0.00, 0.00]	0.04 **[−**0.09, 0.17]	0.06 **[−**0.07, 0.19]	**−**0.01 **[−**0.12, 0.09]	**−**0.00 **[−**0.11, 0.10]	**−**0.02 **[−**0.13, 0.10]	0.02 **[−**0.09, 0.13]	0.01 **[−**0.11, 0.13]	0.03 **[−**0.08, 0.14]
RHR over time ($\beta$_*TimeRHR*_)	0.00 **[−**0.00, 0.00]	**−**0.05 **[−**0.19, 0.09]	0.06 **[−**0.09, 0.20]	0.00 **[−**0.10, 0.10]	**−**0.02 **[−**0.13, 0.08]	0.02 **[−**0.10, 0.14]	**−**0.04 **[−**0.15, 0.07]	**−**0.01 **[−**0.13, 0.11]	**−**0.03 **[−**0.14, 0.09]
TST on mood ($\beta$_*TSTM*_)	0.00 **[**0.00, 0.00]	0.03 **[−**0.10, 0.18]	0.04 **[−**0.11, 0.18]	**−**0.03 **[−**0.13, 0.08]	0.01 **[−**0.10, 0.13]	**−**0.01 **[−**0.13, 0.11]	**−**0.01 **[−**0.12, 0.10]	0.01 **[−**0.12, 0.14]	**−**0.02 **[−**0.15, 0.10]
TST on RHR ($\beta$_*TSTRHR*_)	**−**0.00 **[−**0.00, 0.00]	0.07 **[−**0.07, 0.21]	0.01 **[−**0.14, 0.15]	**−**0.06 **[−**0.16, 0.05]	0.05 **[−**0.06, 0.15]	0.01 **[−**0.12, 0.13]	**−**0.08 **[−**0.18, 0.04]	**−**0.05 **[−**0.18, 0.08]	**−**0.05 **[−**0.17, 0.07]

*CI* Credibility interval. *TST* prior night’s total sleep time. *Group 1 (reference)* enhanced personalized feedback (EPF) only. *Group 2* Headspace only. *Group 3* Headspace + EPF. *Group 4* SilverCloud only. *Group 5* SilverCloud + EPF.

In the conditional steps model (Table 2), baseline depression (estimate = −0.22; small-medium effect) and baseline anxiety (estimate = −0.13; small effect) were inversely associated with daily mood. Baseline depression was inversely associated with daily mood autoregression (estimate = −0.13; small effect). Higher baseline depression was associated with more variability in daily mood (estimate = 0.14; small effect).

In the conditional RHR model (Table 4), baseline depression (estimate = −0.21; small-medium effect) and baseline anxiety (estimate = −0.14; small effect) were inversely associated with daily mood. Higher baseline depression was associated with more variability in daily mood (estimate = 0.13; small effect).

### Fitbit measures and mood

#### Daily steps

**Fig. 1:**
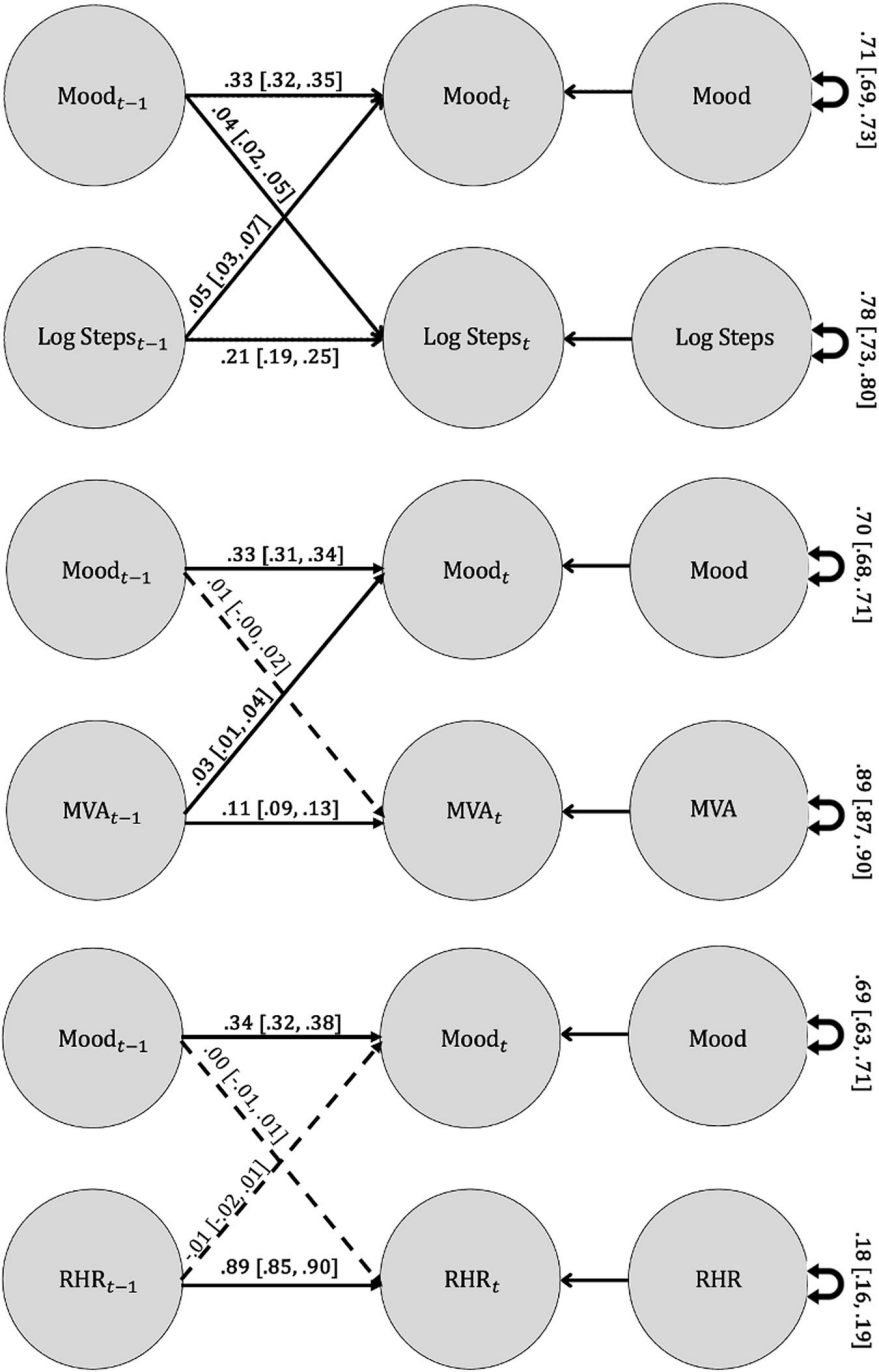
DSEM path models. t time, MVA minutes very active, RHR resting heart rate. Solid lines represent credible paths. Coefficients and 95% credibility intervals are presented.

In the unconditional model, our analysis revealed a bidirectional relationship (cross-lagged association) between mood and daily steps (Fig. 1). When a person reported a mood score above their own average, they tended to walk more steps the next day (estimate = 0.04; small effect). Conversely, on days when individuals took more steps, they generally reported better mood the subsequent day (estimate = 0.05; small effect). Notably, because daily steps were log-transformed, the model coefficients reported here represent effects on a proportional scale. For example, the estimate of 0.04 for mood predicting next-day daily steps means that a 1-SD increase in mood is associated with a 0.04-SD increase in log(steps) (see Supplement Appendix 3 for details on DSEM standardization). Translating this into a specific percent increase in raw step count depends on the scale of log(steps) for each individual. However, to illustrate how the log transformation translates to percent change: a 0.10-unit increase in log(steps) corresponds approximately to a 10.5% increase in steps (since exp(0.10) ≈ 1.105, meaning steps are multiplied by 1.105, or increased by 10.5%). In the other direction, the estimate of 0.05 for daily steps predicting next-day mood means that a 1-SD increase in log(steps) is associated with a 0.05-SD increase in mood. As an example, this means a real-life change such as doubling one’s daily steps predicts approximately a 0.03-SD increase in next-day mood (since (log(2) ≈ 0.693), and 0.693 × 0.05 ≈ 0.03).

Male sex (estimate = 0.22; small-medium effect) and younger age (estimate = −0.13; small effect) were both associated with higher average step counts (Table 2). Participation in randomization group 4 (SilverCloud) predicted a weaker association between mood and next-day steps (estimate = −0.22; small-medium effect) compared to participants in group 1 (EPF).

#### Very active minutes

In the unconditional model (Fig. 1), when a person had more very active minutes compared to their own average, they reported better mood the following day (estimate = 0.03; small effect), but not vice versa. In the conditional model (Table 3), male sex appeared as a credible moderator in the non-credible cross-lag between mood and next-day very active minutes (estimate = 0.91; large effect). Male sex (estimate = 0.39; medium-large effect) was associated with increased very active minutes on average. Both male sex (estimate = 0.10; small effect) and younger age (estimate = −0.11; small effect) were associated with a stronger autoregressive effect of very active minutes. Additionally, both male sex (estimate = 0.26; medium effect) and younger age (estimate = −0.13; small effect) were associated with greater day-to-day variability in very active minutes. Attempts to fit a conditional model with baseline PHQ-9 and GAD-7 were unsuccessful due to non-convergence. In a parallel model without randomization groups included, PHQ-9 and GAD-7 did not contribute to the prediction of very active minutes and were therefore removed from the final model.

#### Resting heart rate

The unconditional model with RHR did not identify any credible cross-lagged relationships with mood (Fig. 1). In the conditional model (Table 4), younger age emerged as a credible moderator in the non-credible cross-lag between mood and next-day RHR (estimate = −0.24; small-medium effect). Higher baseline anxiety was associated with more variability in daily RHR (estimate = 0.15; small effect). Younger age was associated with a stronger autoregressive effect of RHR (estimate = −0.15; small effect).

#### Total sleep time (TST)

In all conditional models (daily steps, very active minutes, RHR; Tables 2–4), the effect of prior night’s TST on daily mood was not credible.

#### Sensitivity analyses

In response to reviewer comments, two sensitivity analyses were conducted: one restricted to female participants and one restricted to data collected after COVID-19 vaccines became widely available (July 2021 onward). In both cases, the overall pattern of credible and non-credible paths remained the same as in the primary analyses, suggesting that results were not unduly influenced by sex composition or peak COVID pandemic period.

## Discussion

The present investigation examined day-to-day dynamics between physical activity and fitness—as measured by daily steps, very active minutes, and RHR—and daily mood in individuals with BDs. We hypothesized that within-person increases in daily steps and very active minutes would be bidirectionally associated with better mood, while within-person decreases in RHR would be associated with better mood.

Consistent with our hypothesis, we observed a bidirectional relationship between daily steps and mood: when individuals took more steps relative to their own average, they tended to report better mood the following day, and when mood was higher than one’s average, steps tended to increase the next day. This supports prior research [6, 13, 14], but differs from Merikangas et al. [15], who found activity predicted mood but not vice versa [15]. By disaggregating physical activity into step count and very active minutes in a large, longitudinal BD sample, the present study refines and extends past findings, showing activity intensity may influence whether reciprocal mood-activity associations emerge. Although effect sizes were small, they highlight the potential impact of physical activity on daily mood regulation in BD, suggesting a feedback loop where improved mood leads to more steps, further enhancing mood. These findings offer tangible implications for clinical practice, suggesting that even low-intensity, day-to-day changes in steps could help to sustain beneficial cycles of mood and physical activity in treatment.

We also found that having more very active minutes compared to one’s own average was associated with better mood the following day; however, this relationship was not bidirectional, possibly due to low incidence of very active minutes in our sample (median=5.3 min). However, this finding aligns with Walsh et al. [16], who observed that increased vigorous activity was associated with reduced next-day depressive symptoms [16]. Our study supports and extends these findings by explicitly testing bidirectionality and demonstrating a unidirectional effect in a substantially larger BD sample over a longer period. Alongside our daily steps findings and prior work [6, 13], these results reinforce the role of physical activity in BD symptoms, but suggest very active minutes may not contribute to a positive feedback loop in the same way as daily steps. Therefore, interventions aiming to increase high-intensity exercise may require more support to sustain adherence over time, while lower-intensity activities like walking may be more accessible and sustainable in fostering positive feedback loops between exercise and mood in the real world.

RHR analyses did not reveal any credible cross-lagged relationships with mood, possibly reflecting both cardiovascular change timescales and Fitbit’s limited RHR measurement precision [40]. In conditional models, age was inversely associated with the autoregression effect of RHR, suggesting that daily RHR was more stable from day to day among younger participants. This supports prior research in non-BD populations showing that autonomic regulation patterns change across the lifespan [41]. Additionally, participants with higher baseline anxiety showed increased variability in daily RHR, which, when considered alongside the well-established association between anxiety and reduced HRV [42], may reflect differences between beat-to-beat HRV and day-to-day RHR averages. To clarify the nature and effects of autonomic dysregulation in BD, future studies could incorporate HRV alongside RHR measures.

Conditional models showed that male sex and younger age moderated the relationship between physical activity and mood in BD. On average, male sex was associated with increased very active minutes, and both male sex and younger age were associated with higher daily step counts, stronger autoregressive effects of very active minutes, and increased variability in very active minutes. These results align with past research showing that activity decreases with age [43], and that, among individuals with BDs, aging is associated with increased depressive symptoms (associated with lower activity levels) rather than mixed or (hypo)manic symptoms [44]. It is important to note regarding sex-based findings that female participants comprised the majority of the sample (74.6%), which may have biased effect sizes in male participants. Nonetheless, these findings indicate there may be sex differences in the dynamics between mood and activity in BDs, and replication in more balanced samples would help to clarify these differences.

Focusing on day-to-day within-person effects provides novel insights into how even modest increases in activity could support mood stability, suggesting that low-burden interventions (e.g., gradually increasing daily steps) could be effectively integrated into broader clinical care. One of the most compelling aspects of increasing daily steps as an adjunctive intervention is its accessibility; it is straightforward, low-burden, and easy to titrate. Wearable technology could enable easy monitoring and adjustment of activity levels, which could offer clinicians the potential for a personalized, feasible, and dynamic treatment approach [45]. Additionally, wearable data could positively influence treatment outcomes even independently of clinician involvement; if patients observe that increases in their daily steps correlate with improvements in their mood, they may feel more intrinsically motivated to maintain higher activity levels, thereby reinforcing the positive feedback loop.

Despite this study’s strengths, several limitations warrant acknowledgement. First, the present study measured daily mood using a single self-report item, which differs from more symptom-specific assessments of depression and (hypo)mania. While much prior research supports the validity of such single-item daily mood measures in BD [34, 46, 47], it is possible these measures do not capture the full complexity of mood states. Next, a limitation is the absence of several covariates in the conditional DSEM models, including specific BD diagnoses, medication use, and psychotherapy treatment, due to an insufficient level of detail in the EHR. Because of this lack of specificity (e.g., chart diagnoses were generalized across all types of BD), we could not confidently categorize or include these variables in our analyses. Future studies would benefit from incorporating more precise data on BD diagnosis, medication use, and psychotherapy treatment to better account for potential moderating or mediating effects. An additional limitation is that the analyses did not account for seasonal or cyclical trends; because participants contributed varying numbers of days and seasons of data, and because many participants had incomplete annual coverage, systematic examination of seasonality was not possible. Lastly, this study’s sample was highly homogenous. Although reflective of the population of the recruitment catchment area, the study sample was predominantly female and White, which may limit the generalizability of the findings. Replicating this study in more diverse populations would help improve generalizability.

The findings of this study provide many avenues for future research. First, exploring the impact of different types of physical activity (e.g., structured exercise versus incidental activity) could provide more nuanced insights for developing interventions. It could also be valuable to explore the minimum threshold of physical activity necessary to achieve mood benefits, as well as the potential for diminishing returns at higher levels of activity. Future work could explore other sleep- and circadian-related variables in their relation to daily mood, including light exposure and chronotype. Lastly, future studies could examine whether RHR moderates the relationships between physical activity and mood, or instead use HRV rather than RHR. These insights could help refine clinical interventions that are effective and accessible.

In sum, this study replicates and extends prior research by suggesting that physical activity confers small but credible improvements in mood on a day-to-day basis in a large longitudinal sample of individuals with BDs. While these findings provide evidence that physical activity is but one factor among many that impacts mood health, they nonetheless underscore how small, real-world behavioral changes can help in the daily management of BD. Future research should explore which types and amounts of activity are most beneficial, and how factors like specific BD diagnosis and treatment impact these dynamics.

### Citation diversity statement

The authors have attested that they made efforts to be mindful of diversity in selecting the citations used in this article.

## Supplementary information

Supplemental Materials

## Data Availability

Per University of Michigan policy, data cannot be shared without a fully executed data use agreement (DUA). For interest in accessing data used in this publication please contact the corresponding author who can facilitate communication with relevant parties to initiate DUA upon reasonable request.
