# Supplementary material for: Integrating active and passive digital phenotyping to study the temporal dynamics between physical activity and mood in bipolar disorders
Source: NPP Digit Psychiatry Neurosci. 2025 Dec 2;3:30. doi: 10.1038/s44277-025-00050-z (PMC12669782; doi:10.1038/s44277-025-00050-z)
Supplement: Supplementary file 1 — Supplemental Materials [file 44277_2025_50_MOESM1_ESM.docx]

Table of Contents

[Figure S1: Zero-Order Correlations at the Between- and Within-Person Level 2](#_Toc213920903)

[Figure S2: DSEM Path Models for Female-Only Sensitivity Analyses 3](#_Toc213920904)

[Figure S3: DSEM Path Models for Peak COVID-19 Period Sensitivity Analyses 4](#_Toc213920905)

[Table S1: Participant Demographics at Study Entry (*N* = 252) 5](#_Toc213920906)

[Table S2: Per-Person Baseline Depression and Anxiety and Daily Fitbit and Mood Descriptives 6](#_Toc213920907)

[Table S3: Observation-Level Baseline Depression and Anxiety and Daily Fitbit and Mood Descriptives 7](#_Toc213920908)

[Table S4: Daily Fitbit Measures Descriptives by Sex and Age Group 8](#_Toc213920909)

[Appendix 1: PROMPT Study Intervention Details 9](#_Toc213920910)

[Appendix 2: Data and DSEM Pre-Processing 17](#_Toc213920911)

[Appendix 3: DSEM Modeling 18](#_Toc213920912)

### Figure S1: Zero-Order Correlations at the Between- and Within-Person Level


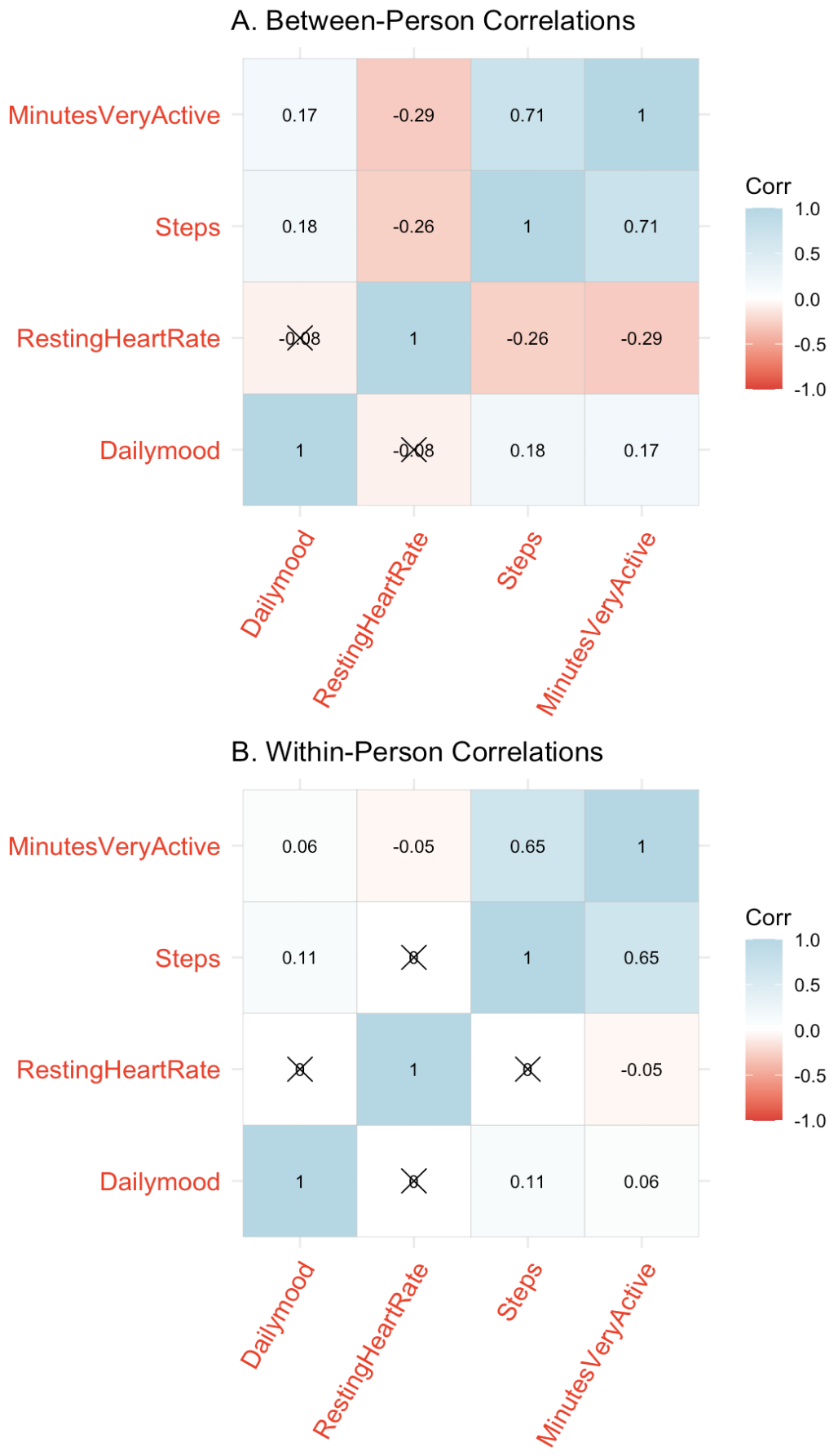


Note. Top (A) represents between-person correlations; bottom (B) represents within-person correlations. Blue shades represent positive associations; red shades represent negative associations. An “X” denotes a correlation that is not statistically significant (*p* ≥ .05).

### Figure S2: DSEM Path Models for Female-Only Sensitivity Analyses


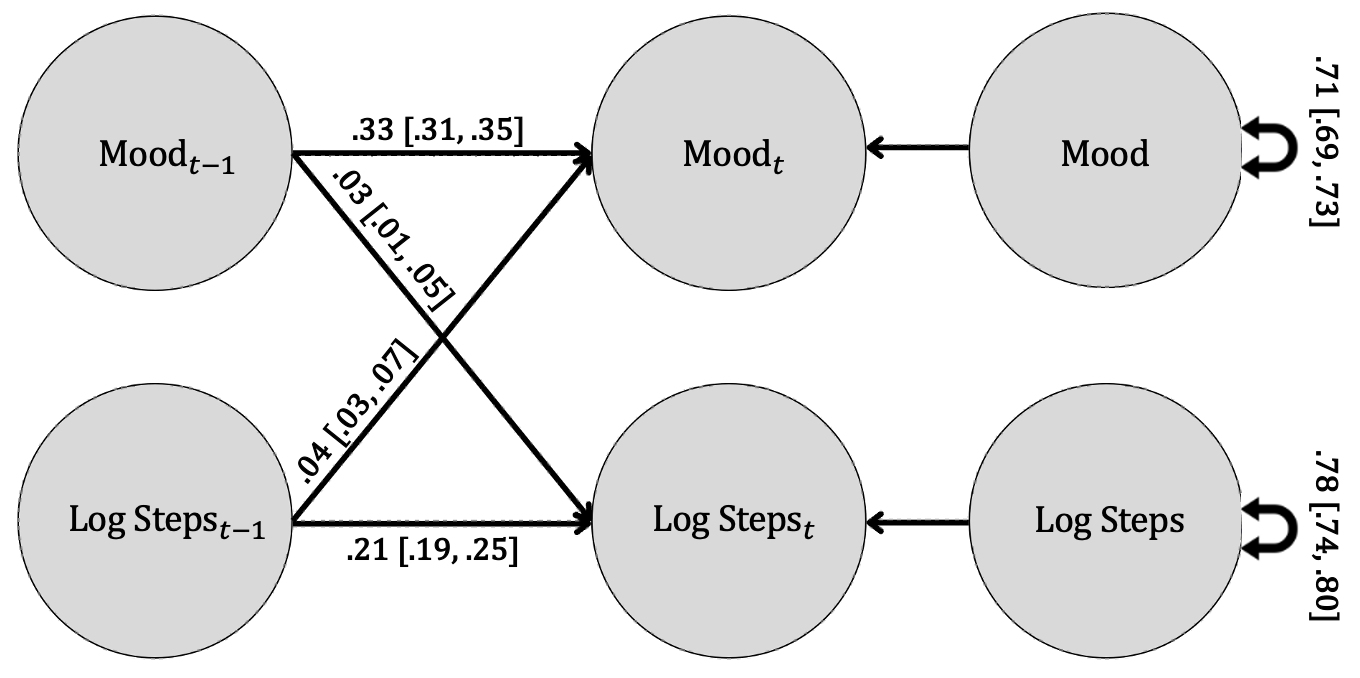


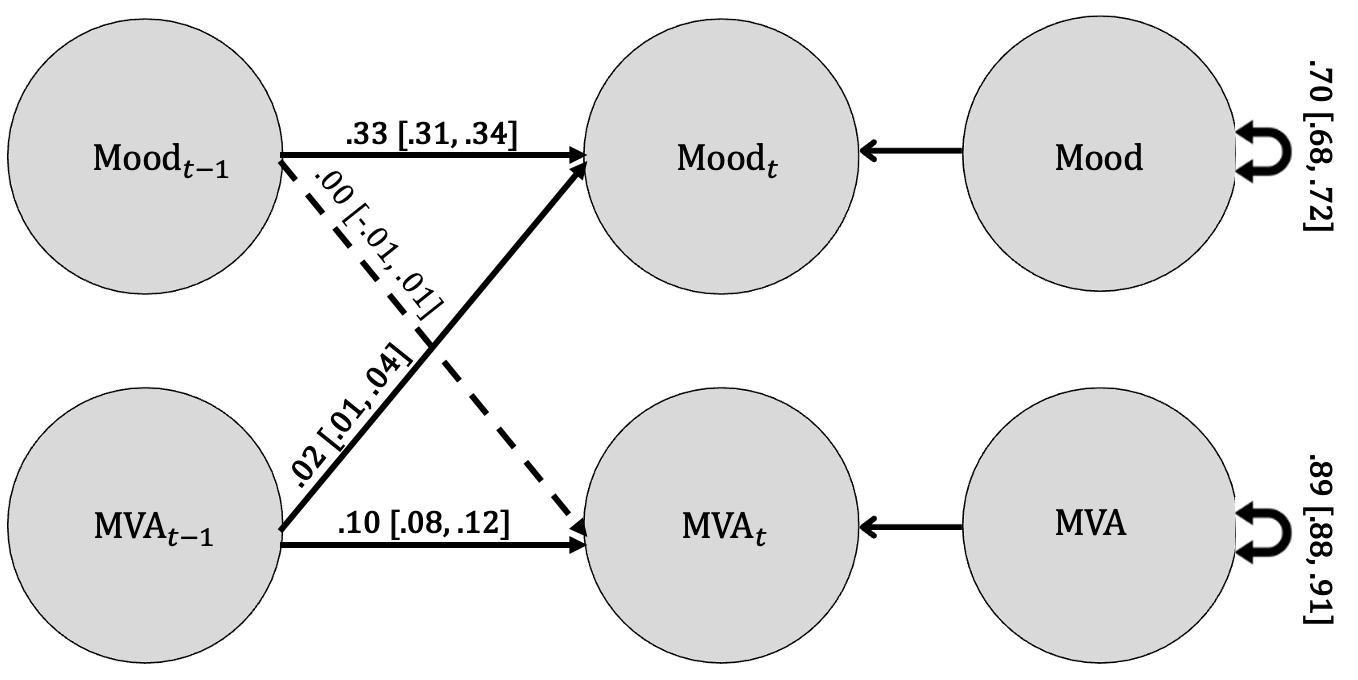


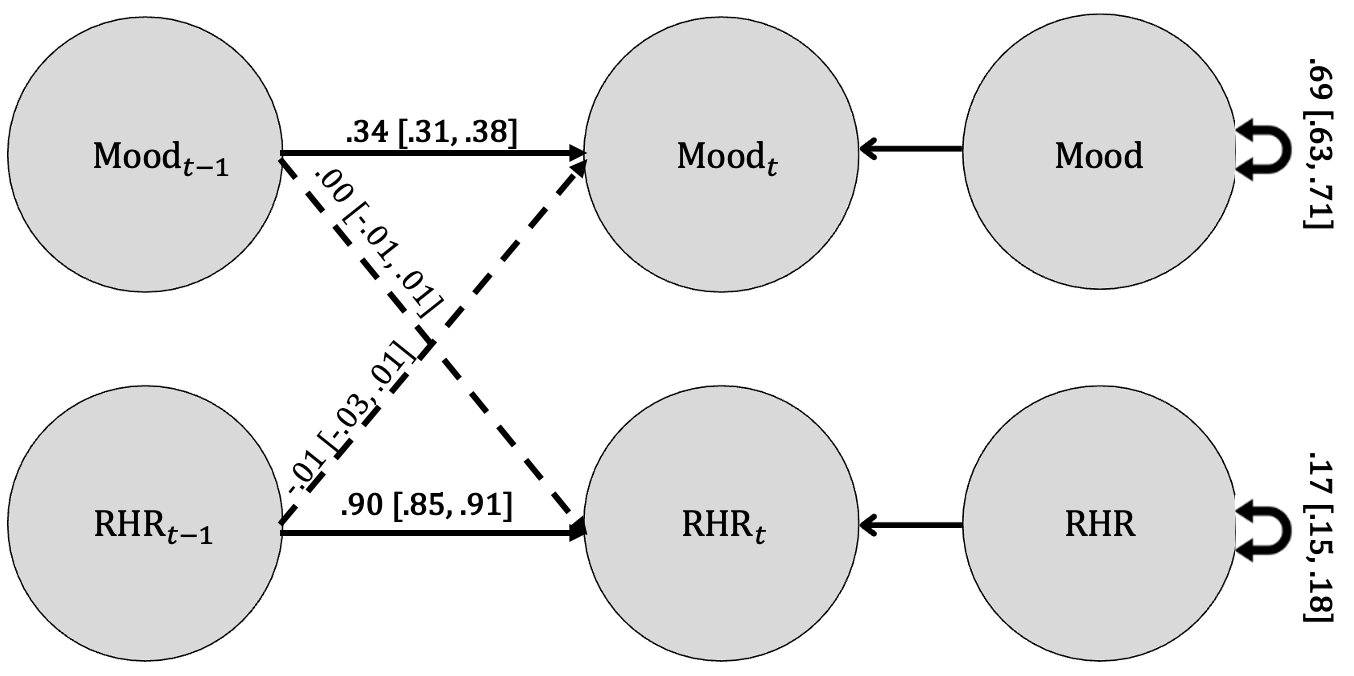


### Figure S3: DSEM Path Models for Peak COVID-19 Period Sensitivity Analyses


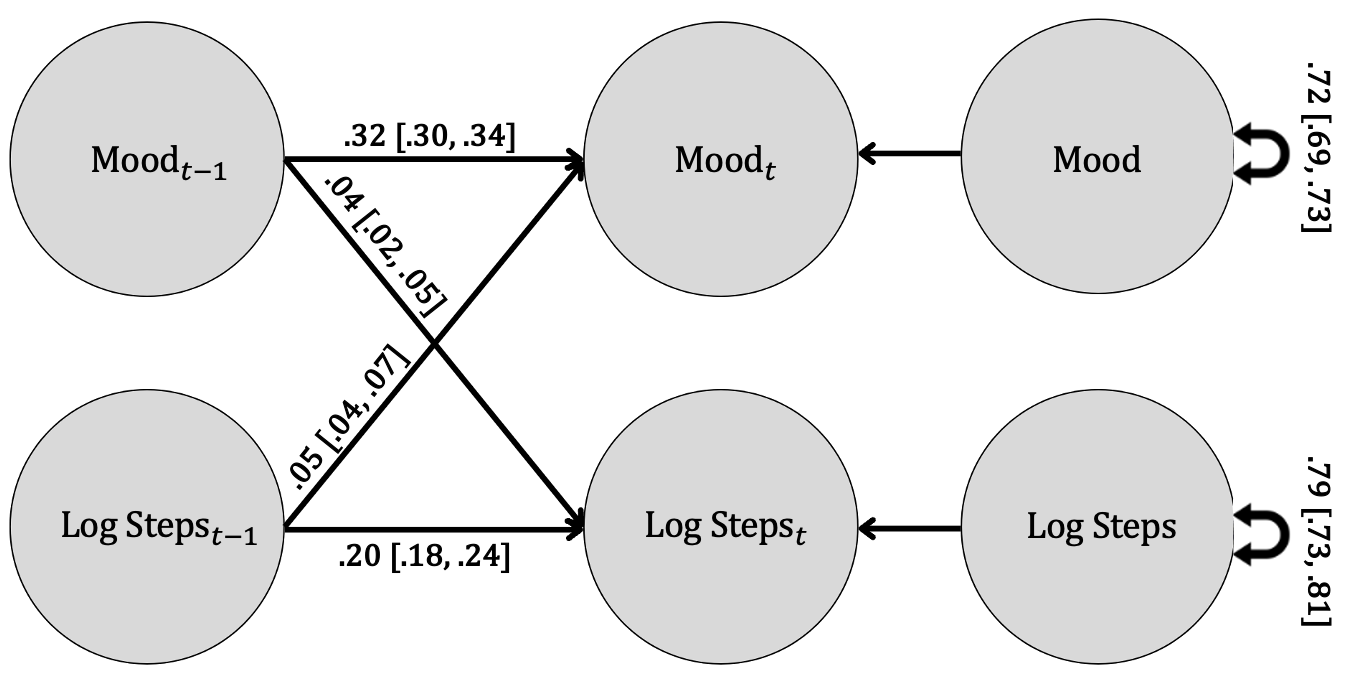


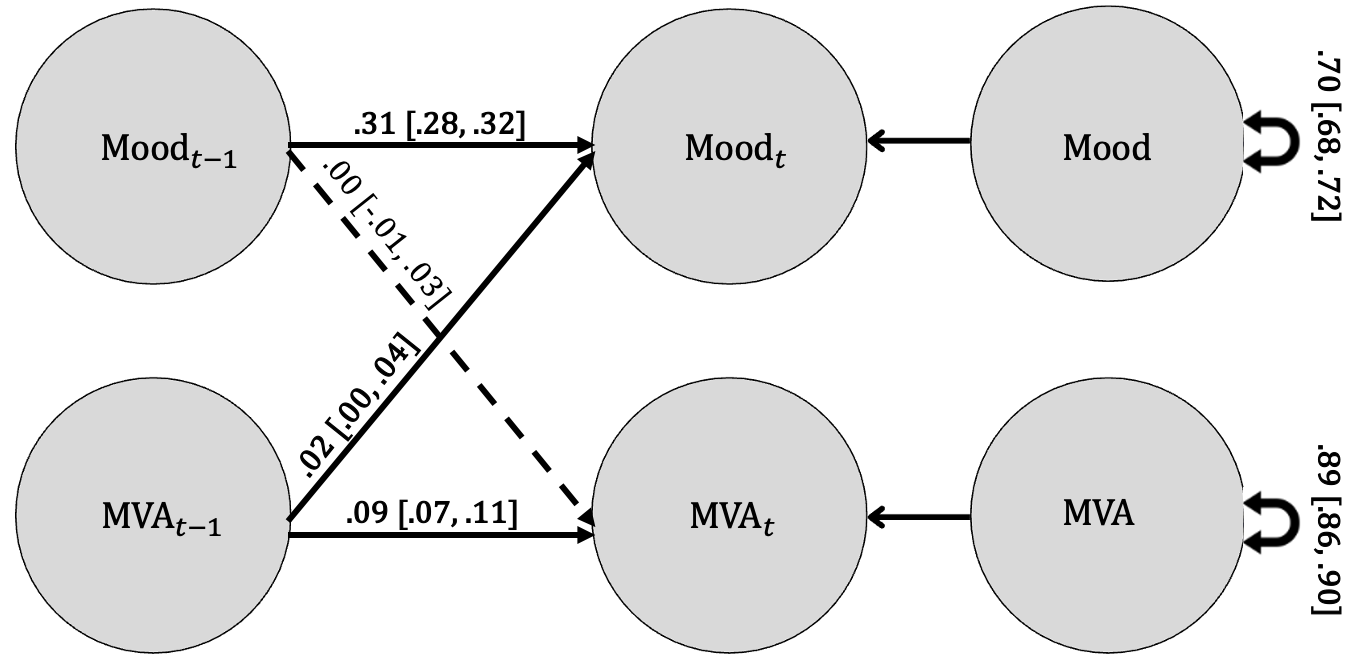


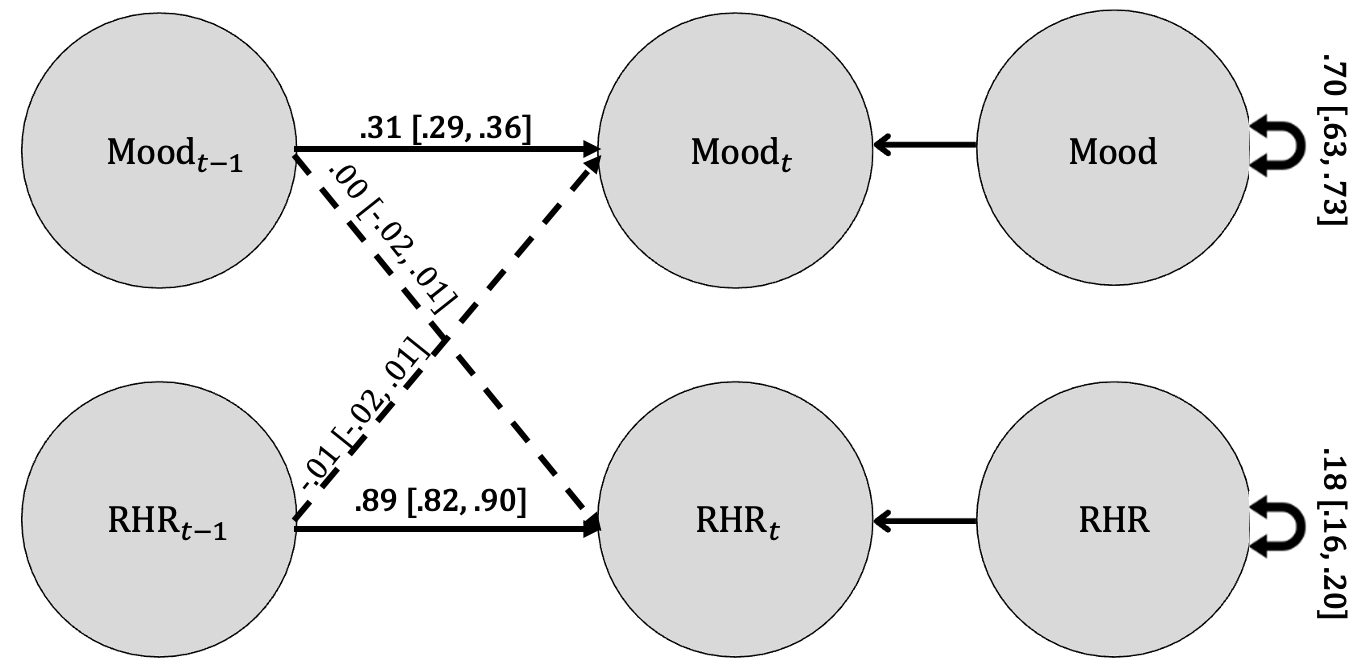


### Table S1: Participant Demographics at Study Entry (*N* = 252)

| **Characteristic** | ***n* (%) or M (SD)** | **Median** | **Range** |
| --- | --- | --- | --- |
| **Sex** |  |  |  |
| Female | 74.6% |  |  |
| Male | 25.4% |  |  |
|  |  |  |  |
| **Age (years)** | 40.37 (14.45) | 37.12 | 18.43 – 76.12 |
|  |  |  |  |
| **Race** |  |  |  |
| White | 79% |  |  |
| Black or African-American | 7.1% |  |  |
| Asian | 5.2% |  |  |
| American Indian or Alaska Native | 2.8% |  |  |
| Other | 2.8% |  |  |
| Patient Refused | 3.2% |  |  |
|  |  |  |  |
| **Ethnicity** |  |  |  |
| Non-Hispanic or Latino | 87.7% |  |  |
| Hispanic or Latino | 7.9% |  |  |
| Unknown | 1.2% |  |  |
| Patient Refused | 3.2% |  |  |

*Note*. Values are presented as *n* (%) for categorical variables and as mean (SD) for continuous variables.

### Table S2: Per-Person Baseline Depression and Anxiety and Daily Fitbit and Mood Descriptives

| **Variable** | **M (SD)** | **Median** | **Range** |
| --- | --- | --- | --- |
| Baseline PHQ-9 | 13.21 (6.45) | 13.00 | 0.00 – 27.00 |
| Baseline GAD-7 | 11.62 (5.96) | 11.00 | 0.00 – 21.00 |
| Step count | 5,984.31 (3,239.42) | 5,444.51 | 70.12 – 19,812.97 |
| Resting heart rate | 70.99 (8.73) | 70.16 | 52.30 – 94.15 |
| Mood | 6.00 (1.42) | 6.08 | 1.11 – 9.76 |
| MVA | 11.24 (14.58) | 5.30 | 0.00 – 83.14 |

*Note*. MVA = minutes very active.

### Table S3: Observation-Level Baseline Depression and Anxiety and Daily Fitbit and Mood Descriptives

| **Variable** | **M (SD)** | **Median** | **Range** |
| --- | --- | --- | --- |
| Baseline PHQ-9 | 13.01 (6.44) | 13 | 0 – 27 |
| Baseline GAD-7 | 11.36 (5.95) | 11 | 0 – 21 |
| Step count | 5,912.46 (4,630.49) | 4,818 | 4 – 77,554 |
| Resting heart rate | 70.63 (9.38) | 70 | 44 - 107 |
| Mood | 6.12 (1.90) | 6 | 1 - 10 |
| MVA | 11.25 (24.59) | 0 | 0 - 404 |

*Note*. MVA = minutes very active.

### Table S4: Daily Fitbit Measures Descriptives by Sex and Age Group

| **Sex** | **Age Group** | ***n*** | **Steps M (SD)** | **RHR M (SD)** | **MVA M (SD)** |
| --- | --- | --- | --- | --- | --- |
| Female | 18–34 | 91 | 5945.98 (2840.65) | 71.48 (8.56) | 9.71 (11.6) |
| Female | 35–64 | 87 | 4983.28 (2672.22) | 71.8 (9.1) | 6.36 (8.69) |
| Female | 65+ | 10 | 3453.68 (1481.55) | 68.75 (8.89) | 2.25 (2.09) |
| Male | 18–34 | 22 | 8744.84 (4268.64) | 66.9 (6.48) | 26.38 (20.26) |
| Male | 35–64 | 36 | 7510.32 (3584.36) | 71.21 (9.24) | 18.81 (20.06) |
| Male | 65+ | 6 | 6020.31 (2782.22) | 69.19 (7.65) | 19.32 (16.72) |

*Note*. M = mean. RHR = resting heart rate. MVA = minutes very active.

### Appendix 1: PROMPT Study Intervention Details

**RESEARCH DESIGN AND METHODS**

This study will have **two phases**, each with different study designs:

**Phase 1** will cover the period while individuals are waiting for care. This phase includes measuring baseline (i.e., pre-clinic) characteristics, and for some patients, receiving Mobile Health (mHealth) interventions.

**Phase 2** begins when a participant has a first appointment for outpatient mental health care, and uses a cohort study design for measuring outcomes associated with clinic-based treatments.

***Phase 1***

Patients with scheduled intake mental health appointments, pre-screening mental health appointments, on the waitlist/referral list, or identified as a potential patient seeking mental health care at Michigan Medicine or University Health Services will be recruited to participate in the study. Patients can be recruited at any time between the appointment being scheduled to up to one month following their intake appointment.

Participants identified with moderate to severe depression based on baseline survey results will be assigned to group one. Participants not identified with moderate to severe depression will be randomly assigned to one of **three groups**:

1. Enhanced Feedback + Standard Feedback (**EF+SF**): Participants will receive enhanced feedback from the MyDataHelps study app in addition to standard feedback from the Apple Watch health app or Fitbit activity tracker. Participants identified with moderate to severe depression, based on a PHQ-9 scores greater than or equal to 10, will be assigned to EF+SF group.
2. App-Based Intervention + Standard Feedback (**App+SF**): Participants will receive an app-based intervention in addition to standard feedback from the Apple Watch health app or Fitbit activity tracker
3. App-Based Intervention + Enhanced Feedback + Standard Feedback (**App+EF+SF**): Participants will receive an app-based intervention, enhanced feedback from the MyDataHelps study app, and standard feedback from the Apple Watch health app or Fitbit activity tracker

As part of the enrollment process, participants will be guided by a study team member through **four processes**:

1. **Download Study App:** Participants will download the study app, MyDataHelps, on their smartphone. MyDataHelps is a commercially available application developed by CareEvolution. This app serves as a vehicle for consent, data collection, delivery of all study questionnaires, automated notifications, and reminders. The app will also receive data from the Health app and study devices. Participants will be asked to complete daily mental health symptom assessments via the app throughout the duration of their enrollment in the study. The app will also present enhanced feedback on participant progress via a personalized “dashboard.” If participants are utilizing an Apple Watch with their existing iPhone, they must enable MyDataHelps to read out the data fields in the Health app. This will be made clear to participants during the consent process, so a participant can decide if they are still interested in participating.
2. **Receive Device:** Participants will be provided with an Apple Watch or Fitbit to sync with their smartphone. Participants with iPhones will be provided with Apple Watches or Fitbits and participants with Androids will be provided with Fitbits, or similar smart watches and activity trackers for both types of phones. This device will be worn throughout their entire involvement in the study. This will allow for collection of objective data on daily activity, such as steps/physical activity, sleep, and heart rate.
3. **Download App-Based Intervention:** Participants will download additional apps to their smartphones that are designed to address mental health symptoms, based on their group assignment. Participants will be computer-assigned to one of the two app-based interventions (mindfulness via the Headspace app or cognitive behavioral therapy via the SilverCloud app). A study team member will guide them through the process of downloading the app to their phone at that time.
4. **Transfer Smartphone Data:** Participants may need to configure their phone settings and/or download Apple SensorKit for iPhones and the Effortless Assessment Research System (EARS) for Android users. A study team member will guide the participants through the process of connecting Apple Sensorkit or EARS to “MyDataHelps”.

Participants will also be asked for permission for the study team to collect active (e.g., symptom self-reports) and passive (e.g., screen time) data through their smartphone through the duration of the study.

**Surveys**

Participants will be asked to complete four surveys, including a baseline survey at the time of enrollment and three follow-up surveys, which will be designed to complement in-clinic assessments completed as a part of standard outpatient mental health care. To the extent possible, redundancy with in-clinic assessments (i.e., due to the Measurement-Based Care program) will be avoided. The first follow-up survey will occur at the end of 6 weeks post-enrollment, the second at the end of 18 weeks post-enrollment and the third at the end of 12 months post-enrollment.

Depending on waitlist length for outpatient mental health services, there may be variation in whether participants have engaged with clinical care by the time they complete their follow-up survey(s). Surveys will be administered by the predetermined schedule regardless of whether participants have engaged with care. The rationale for this decision, rather than scheduling follow-up surveys to coincide with the initiation of outpatient care:

1. There is a possibility that participants may never engage with outpatient care, and these non-engagers would never receive follow-up surveys
2. Waitlist time often varies between recruitment sites (i.e., UHS tends to have a shorter waitlist for care), which would create measurement biases (e.g., UHS patients may consistently engage in care with less time to engage with the app-based intervention)
3. Participants may be receiving other mental health care (e.g., via primary care), making it less important to time follow-up surveys with the initiation of outpatient mental health services
4. The existing timeline ensures all participants assigned to the groups receiving app-based interventions have received a reasonable ‘dose’ of the intervention prior to assessment
5. The existing timeline ensures that measurement will take place at generally the same time frame for all participants

*Summary of tasks that participants will be asked to complete during Phase 1:*

- Complete daily mental health symptom assessments via the MyDataHelps app
- Wear assigned device (Apple Watch or Fitbit) regularly for objective data collection
- Engage with assigned app-based intervention (Headspace or SilverCloud) regularly
- Complete baseline assessment via the MyDataHelps app
- Complete 6-week follow-up survey
- Complete 18-week follow-up survey
- Complete 12-month follow-up survey
- Provide a biological specimen

***Phase 2***

Phase 2 begins when participants establish care with a clinical provider at Michigan Medicine Outpatient Psychiatry or UHS. In Phase 2, participants will engage with outpatient mental health care services as part of routine care, and the study will continue to gather both active and passive data on participants’ symptoms and activity, similar to Phase 1. Participants will continue to have access to their assigned app-based intervention while concurrently receiving outpatient mental health services. The study will collect data from participants’ phones and apps for up to one year following the initiation of clinical care or until participants have completed their 12-month follow-up survey. Participants may still remain enrolled in the study if they cease engagement with outpatient mental health care services during this phase.

*Summary of tasks that participants will be asked to complete during Phase 2:*

- Complete daily mental health symptom assessments via the MyDataHelps app
- Wear assigned device (Apple Watch or Fitbit) regularly for objective data collection
- Engage with assigned app-based intervention (Headspace or SilverCloud), if applicable
- Complete 6-week follow-up survey
- Complete 18-week follow-up survey
- Complete 12-month follow-up survey
- Provide a biological specimen (if not yet completed during Phase 1)

**Screening and Recruitment**

Participant screening and recruitment will be managed by study staff. All study team members are required to complete team/study training and all appropriate regulatory training and disclosures.

The study will recruit 4,500 participants via patients at Michigan Medicine and student patients at University Health Service (UHS). Potential study participants will include adult patients (age 18+) that are scheduled for an intake mental health appointment, pre-screening mental health appointment, on the waitlist/referral list, or identified as a potential patient seeking mental health at either Michigan Medicine or UHS. These appointments are generally scheduled with a 3-10 week waiting period. Patients will be invited to participate in the study as soon as possible after scheduling their appointment in order to best maximize participation during their full waiting period.

A waiver of HIPAA authorization is requested in order for study staff to:

1. Receive daily lists of patients for whom a mental health intake appointment has been scheduled at each recruitment site
2. Access the Michigan Medicine and UHS electronic health records to confirm eligibility of listed patients and obtain contact information

Study staff will employ the following **recruitment strategies** to contact, screen, and enroll potential participants:

1. At the time of scheduling their intake mental health appointment, patients may be informed by call center staff that they may be contacted about a new study.
2. Daily lists of newly scheduled appointments and individuals on the waitlist/referral list will be generated by the Michigan Medicine Department of Psychiatry call center, UHS scheduling, or from the Research Data Warehouse (RDW) and provided to study staff.
3. Using existing Research Data Warehouse information, a patient is prospectively identified as someone who meets the study criteria during the screening process. Potentially eligible patients may be contacted by email/text message/phone call to introduce the study, with an invitation to contact the study team by phone or email if interested in participating.
4. Prior to contacting patients by phone/text message/phone call, study staff will complete preliminary screenings via listed patients’ electronic health records to determine whether basic, objective inclusion criteria are met (e.g., scheduled outpatient mental health appointment, age 18+).
5. Study staff will follow up with a phone call/text message/phone call within 2 business days of the patient’s appointment scheduling to further describe the study and assess interest.
6. Patients identified by preliminary screenings as potentially eligible may be sent a standardized study letter by mail to introduce the study, with an invitation to contact the study team by phone/text message/email if interested in participating.
7. Interested patients will be asked additional screening questions to determine eligibility (e.g., owning a smartphone, willingness to keep study setup configured on smartphone for duration of study). Eligibility screening questions may be administered via phone contact with the potential participant or within the MyDataHelps app prior to consent.
8. Interested and eligible patients will be invited to the study via the RKStudio study portal email system and directed to download the MyDataHelps study app on their smartphone. Account creation within the app will facilitate participant access to the informed consent document and enrollment in the study.

Study staff will conduct a preliminary screening via the electronic health record for eligibility, and will then contact potentially eligible participants by phone or email to offer a meeting at their first appointment to complete the study enrollment processes. If the patient does not show for the intake appointment, study staff will follow up by phone to offer further information and screening for the study. These participants may also opt to complete the study processes remotely (e.g., phone, app) or in a separate agreed-upon location (e.g., in the community), if desired.

**Consent**

Informed consent will be conducted within the MyDataHelps app via an eConsent. A waiver of documentation of consent is requested to obtain electronic consent for this study.

If a patient is interested in participating, a study team member will invite them to the study using the RKStudio study portal email system. Participants will be directed to download the MyDataHelps app on their smartphone through the Apple or Google Play Store (dependent on smartphone), which will include an IRB-approved study consent module. When a participant opens MyDataHelps, they are first required to register an account using their email, and are subsequently presented with the eConsent form that lays out the process of participation. The patient is asked their name and for a finger-drawn signature. Study personnel will be available by phone or email to answer any questions participants may have about the consent and study. If participants do not complete the informed consent process via the study app and have not indicated that they do not wish to participate in the study, study staff will follow up by phone or email to answer questions or provide assistance. Participants will have the option to download a copy of the consent document or have a copy emailed or mailed to them.

**Enrollment**

Participants will complete several steps prior to enrollment being considered as “completed.”

Participants must complete the following steps to be enrolled in the study:

1. MyDataHelps study app download, installation, and account creation
2. Completion of informed consent
3. Completion of baseline survey via the MyDataHelps study app
4. Download and installation of assigned app-based intervention (Headspace or SilverCloud), if applicable
5. Receipt of device (Apple Watch or Fitbit) and agreement to wear the device regularly. Participants’ devices will be mailed, unless they indicated that they would like to meet with study staff to receive their device in person.
6. Pairing and setup of smartphone, device (Apple Watch or Fitbit), and MyDataHelps study app

Study staff are available by phone or email as necessary to provide technical support to aid participants in downloading apps, completing informed consent, or setting up their devices. Participants may also opt to meet with study staff in person, as described above.

Participants will be offered to complete consenting and enrollment steps remotely (via telephone with a study team member and on their own following their download of the MyDataHelps study app). If further assistance is needed, participants may opt to meet recruitment staff in person for support with downloading app(s), completing the informed consent process, completing the baseline survey, or picking up and configuring their Apple Watch or Fitbit. Patients who begin the recruitment process online may also opt to meet recruitment staff in person to complete the remainder of the process (e.g., informed consent, enrollment, downloading apps). In-person meetings may take place in a designated study space at Michigan Medicine or UHS or at an agreed-upon location in the community (e.g., coffee shop, campus library).

**Inclusion Criteria**

1. Must have a scheduled intake mental health appointment, pre-screening mental health appointment, on the waitlist/referral list, or identified as a potential patient seeking mental health care at Michigan Medicine or University Health Services
2. Age ≥ 18 years
3. Must have daily access to a smartphone to use for the study (iPhone 5s or later, depending on Apple Watch version available, or Android smartphone compatible with relevant Fitbit model and study app)
4. Understands English to enable consent and use of the MyDataHelps app and app-based interventions
5. Provide complete, updated contact information upon enrollment to the study
6. Agree to be contacted by study staff during the study
7. Willingness to keep study setup (devices, apps, settings) in required configuration for the duration of the study, and following instructions by study staff if required to update or re-achieve required configuration if required configuration has been lost (e.g., change of phone, app deletion)

**Exclusion Criteria**

1. Deemed unable to provide informed consent (e.g., cognitive inability, guardianship)
2. Eating disorder, unless well controlled, as self-reported by the patient, verified by the research participant's mental health team, or review of medical chart by study team
3. Scheduled outpatient mental health appointment is a pediatric appointment (even if age 18+)
4. Wrist too large or too small to wear an Apple Watch or Fitbit comfortably (Note: bands that are shorter or longer than the standard will be made available to minimize this exclusion)

Those who have known allergies or previous reactions to fluorocarbon-based synthetic rubber, such as contact dermatitis with fluoroelastomer bands primarily used in wrist-worn fitness devices, will be offered an alternate Apple Watch or Fitbit band.

Participants may be excluded at the discretion of the Principal Investigators based on the determination that it is in the participant’s or study staff’s best interest (e.g., to fully ensure participant privacy in the case that a study staff member knows a patient or their family personally).

**Participant Remuneration**

Participants will be remunerated $20 for completing the baseline survey, $20 for the first follow-up survey, $40 for the second follow-up survey, and $50 for the third follow-up survey. Participants could receive a total of $130 for surveys via cash or gift card if they complete all study activities. Participants will be given an Apple Watch or Fitbit to use during their participation in the study and may keep these devices once they have completed the study. The Apple Watch Series 3 is valued at about $250 and Fitbit Charge 3/Inspire HR valued at about $120. Other versions of these devices or similar devices may be given to participants, based on availability. Between completion of surveys and receipt of Apple Watch, Fitbit, or similar devices, total participant remuneration will range from $250 to $380 via cash or gift cards and receipt of mobile health device, dependent on the mobile health devices available and received.

**MHEALTH INTERVENTIONS**

*Standard Feedback (SF)*

All participants will receive the feedback that is standard with the Apple Watch or Fitbit devices. Both sensors provide feedback on activity level, heart rate, and progress toward daily activity goals on the device itself. For the Apple Watch, this also includes feedback from the iPhone’s “Activity” and “Watch” apps. For Fitbit users, this includes feedback in the “Fitbit” app.

*Enhanced Feedback (EF)*

Participants assigned to receive enhanced feedback will receive this feedback from the MyDataHelps study app. This includes varying types of text and visual feedback based on data collected through the app. Feedback will be displayed to participants on a dashboard in the app or delivered via pop-up notifications. Participants will receive a combination of text and visual feedback.

Examples of potential text feedback:

- A summary statement connecting behaviors (e.g. “On Tuesday, your mood was a 9. That night, you slept 6 hours. Your mood was a 7 the next day.”)
- Empathetic feedback about behavioral patterns (e.g. “You had a tough week. On Tuesday, when you were at the hospital for 12 hours, your mood was a 6. What have you done in the past to help your mood when you had a hard day?”)
- A motivational tip (e.g. “It seems like you have had a tough week. Hang in there – you’ve survived worse!”)

Examples of potential visual feedback:

- A graph plotting one data type over time (e.g. hours of sleep for the past week)
- A graph plotting the relationship between different data types over time (e.g. mood and sleep hours for the past month)

We also plan for some feedback to be customized based on subjects’ mood, activity, and sleep data. For example, subjects who slept for less than 6 hours on average in the past week might receive different feedback than those who slept more.

*App-Based Interventions*

Participants assigned to the groups receiving the apps will be asked to install an mHealth app onto their smartphone. The two apps that will be used are Headspace, an app designed to train the user in mindfulness practices, and Silvercloud, an app designed to deliver cognitive behavioral therapy. These apps were selected because of evidence of efficacy and applicability across a wide range of mental health symptoms. Participants receiving apps will be computer-assigned to receive one of the two options

App notifications will encourage participants to use the apps regularly. If user data indicates that a participant is not using the apps, study staff will contact the participant via app notification, phone call, email, letter, or text message. SilverCloud allows for integration of a health coach as a supportive role for increasing engagement, largely through asynchronous messaging. Study staff will be trained in the role of the health coach and will engage with participants through the app to promote app use, personalization, and overall engagement. For both apps, study staff will provide ongoing technical assistance and address any participant barriers to app use.

### Appendix 2: Data and DSEM Pre-Processing

**Data Pre-Processing**

To ensure data quality, the Fitbit data were cleaned and pre-processed before analysis, resulting in 252 individuals with sufficient data for inclusion. For each day per participant, we retained a single data entry based on highest/final step count and ensured Fitbit adherence (e.g., calculating wear time). Across the entire dataset, a total of 32,451entries were recorded. On average, participants contributed 125.92 days of Fitbit data (range = 30–347; median = 105; SD = 79.91). For mood EMA data, participants contributed an average of 128.77 days (range = 30–348; median = 108; SD = 81.15).

***Daily Step Count Selection***

Because Fitbit records multiple lines of data per day (when the user opens the app or syncs their devices), the first step in cleaning was to ensure that each participant had one row of data per day. To do this, we used the row with the final and highest step count of the day, which represents the final data recording of that day. Selecting the data in this way ensured that duplicate or incomplete records were excluded for all variables of interest (steps, RHR, and very active minutes).

***Adherence Criteria: Minimum Wear Time and Data Contribution***

To ensure data quality, the final dataset only included days when the Fitbit was worn for at least 10 hours of waking wear. This decision is in line with best practices^1^ and was intended to filter out days with sporadic or inconsistent Fitbit use, improving the reliability of the final dataset.

Participants were included in the final analysis only if they contributed at least 30 days of valid data (as defined by the above criteria). This threshold was chosen to suit the requirements for DSEM.

**DSEM Pre-Processing**

***Log Transformation of Steps***

The daily steps variable was log-transformed to reduce variability, which was initially too high for effective modeling in DSEM. This transformation helped stabilize variances, reduce skewness, and improve the model’s interpretability. Because daily steps were log-transformed, all coefficients involving daily steps should be interpreted on a proportional scale.

### Appendix 3: DSEM Modeling

Dynamic Structural Equation Modeling (DSEM) decomposes within- and between-person variance using latent mean centering and allows for multiple random effects and their covariances to be modeled simultaneously. DSEM is a Bayesian multivariable multilevel first-order vector autoregressive model estimated in MPlus v8.8. DSEM uses the Kalman filter approach for estimating missing data based on lagged observations and Markov Chain Monte Carlo (MCMC) to estimate posterior distributions. MPlus’ default settings adaptively assess convergence by drawing a minimum of 1,000 samples over two chains (with thinning = 10 and the first half of samples discarded as “burn-in”) and iteratively assessing Potential Scale Reduction values (PSR) 32 for all parameters until they reach an acceptable range (i.e., <1.1). To isolate within-person effects, DSEM automatically applies latent person-mean centering to within-person variables.^2^

Using DSEM, our models result in eight random effects for each individual: mean intensity, autoregressive coefficients, cross-lagged regressions, and innovation variance. Means represent the average of each variable over individuals’ entire time series. Autocorrelations reflect the extent to which a variable is associated with its score at the subsequent time point. Conceptually, a high autocorrelation could mean two things: (1) that when an individual has a deviation from their own average, they are more likely to retain that deviation at the next time point, or (2) their score at one point in time is highly correlated with their score at the next time point (regardless of whether the score itself is high or low). Cross-lags show how much a change in one measure predicts a change in the other measure at the next time point. For example, a significant positive cross-lag between mood at time*_t-1_* and step count at time_t_ indicates that when a person has an increase in mood compared to their average, they are more likely to have an increase in step count at the next time point. Because MPlus standardizes all continuous within-person variables by default in DSEM, all reported regression estimates reflect standardized effects, meaning that the expected change in the outcome (in SD units) is associated with a 1-SD change in the predictor. The innovation variance parameters reflect the leftover variance—that is, how much a person’s scores vary from their mean from one time point to the next, considering the autocorrelation.^2^

Supplement References

1. Chan A, Chan D, Lee H, Ng CC, Yeo AHL. Reporting adherence, validity and physical activity measures of wearable activity trackers in medical research: A systematic review. *Int J Med Inform*. Apr 2022;160:104696. doi:10.1016/j.ijmedinf.2022.104696

2. Asparouhov T, Hamaker EL, Muthén B. Dynamic structural equation models. *Structural Equation Modeling: A Multidisciplinary Journal*. 2018;25(3):359-388. doi:<https://doi.org/10.1080/10705511.2017.1406803>
